# Between ambitions and actions: how citizens navigate the entrepreneurial process of co-producing sustainable urban food futures

**DOI:** 10.1007/s10460-023-10425-7

**Published:** 2023-04-11

**Authors:** Koen van der Gaast, Jan Eelco Jansma, Sigrid Wertheim-Heck

**Affiliations:** 1 Food and Healthy Living Group, Aeres University of Applied Sciences, Arboretum West 98, Almere, 1325 WB The Netherlands; 2 Urban Economics Group (UEC), Wageningen University and Research, Hollandseweg 1, Wageningen, 6706 KN The Netherlands; 3 Environmental Policy Group (ENP), Wageningen University and Research, Hollandseweg 1, Wageningen, 6706 KN The Netherlands; 4 Wageningen Plant Research, Wageningen University and Research, Edelhertweg 1, Lelystad, 8219 PH The Netherlands

**Keywords:** Food, Sustainable, Futuring, Entrepreneurship, Urban agriculture, Almere

## Abstract

Cities increasingly envision sustainable future food systems. The realization of such futures is often understood from a planning perspective, leaving the role of entrepreneurship out of scope. The city of Almere in the Netherlands provides a telling example. In the neighborhood Almere Oosterwold, residents must use 50% of their plot for urban agriculture. The municipality formulated an ambition that over time, 10% off all food consumed in Almere must be produced in Oosterwold. In this study, we assume the development of urban agriculture in Oosterwold is an entrepreneurial process, i.e. a creative (re)organization that is ongoing and intervenes in daily life. To understand how this entrepreneurial process helps to realize sustainable food futures, this paper explores what futures for urban agriculture residents of Oosterwold prefer and deem possible and how these futures are organized in the present. We use futuring to explore possible and preferable images of the future, and to backcast those images to the present day. Our findings show residents have different perspectives of the future. Furthermore, they are capable in formulating specific actions to obtain the futures they prefer, but have trouble committing to the actions themselves. We argue this is the result of a temporal dissonance, a myopia where residents have trouble looking beyond their own situation. It shows imagined futures must fit with the lived experiences of citizens in order to be realized. We conclude that urban food futures need planning and entrepreneurship to be realized since they are complementary social processes.

## Introduction

Increasingly cities are seen as the main locus in making the urban food system more sustainable (Morgan 2010; Morgan and Sonnino 2010; Sonnino 2010; Battersby and Watson 2019; Partzsch et al. 2022). This manifests in municipal governments taking measures, ranging from specific food policies to shared policy pacts with other cities (Moragues-Faus and Morgan 2015; Sibbing et al. 2021). Cities are also transformative spaces where citizens come together to experiment with new urban arrangements. Previous studies show how citizens are brought together to coproduce the future sustainable urban food system in the making (Hebinck et al. 2018; Vervoort and Mangnus 2018; Mangnus et al. 2019). In the burgeoning literature on urban future food systems, the role of planning is thoroughly discussed in relation to the realization of imagined urban food futures (Morgan 2014; Opitz et al. 2015; Sonnino and Coulson 2020). In contrast, the role of entrepreneurship in coproducing urban food systems is seldom explicitly addressed. Even though entrepreneurship is considered a force of social change (Steyaert and Hjorth 2006; Calás et al. 2018) and is known to emerge out of efforts of local communities to organize collectively (Cucchi et al. 2021).

Almere Oosterwold poses an illustrative case. Oosterwold is a relatively new neighborhood in the city of Almere in the Netherlands. Citizens can buy a plot in Oosterwold to build their own house. In return, the new residents of Oosterwold must use 50% of their plot for urban agriculture. The municipality of Almere formulated an ambition for this neighborhood: to produce 10% of total food consumption in Almere over time. In their policy, the municipality implicitly assumes that residents of Almere all are on the same page with regards how urban agriculture could and should develop in the future. Despite having the ambition of sourcing the city of Almere with the produce from Oosterwold, the municipality did not employ a planning strategy for the distribution of the food from residents in Oosterwold to the citizens in the rest of Almere. It is up to the residents to organize this themselves.

We assume the process of organizing urban agriculture in Oosterwold can be characterized as entrepreneurial. We define an entrepreneurial process as ongoing, creative (re)organizing that is always becoming and that intervenes in everyday life (Verduyn 2015). This definition implies entrepreneurship is not separated but part of everyday lived experiences of residents (Steyaert and Katz 2004; Steyaert and Hjorth 2006). Residents of Oosterwold continuously coproduce the future of urban agriculture in their neighborhood as part of their own everyday life. To understand better how this entrepreneurial process helps to realize sustainable food futures, this paper explores what futures for urban agriculture residents of Oosterwold prefer and deem possible and how these futures they imagine are organized in the present.

This study uses ‘futuring’, which refers to a range of methods and techniques that are used to explore more sustainable futures (Hajer and Pelzer 2018; Hebinck et al. 2018; Oomen et al. 2021) We use a combination of visioning, scenario building and backcasting to imagine preferable and possible futures and reason from futures back to actions in the present. This results in facilitating the co-production process of residents of Oosterwold. During the research process, we provided a methodological platform where participants could engage the future and reason back from the future to distill concrete actions and plans. This deliberation helps us to understand how this entrepreneurial process unfolds. In the next section, we introduce the case of Almere Oosterwold and our conceptual take on the relationship between planning, entrepreneurship and the lived experiences of citizens. In our methodological section we explain our approach to futuring. Following, our findings section presents the contrasting future perspectives, and the struggle to organize the future into the present. Lastly, in our discussion we will reflect on what we have learned about the relation between futuring, entrepreneurship and planning in the case of Oosterwold.

## Contextual and conceptual background

### The planning of Almere Oosterwold: a brief history

Almere is the capital of the province of Flevoland in the Netherlands. This province was created by reclaiming land from the sea in the mid-twentieth century, primarily for agricultural purposes. Almere emerged in the seventies to accommodate the increasing population pressure of the nearby city of Amsterdam (Jansma and Wertheim-Heck 2021; van der Gaast et al. 2022a; van der Gaast 2023). The last decade, the city of Almere prioritizes food policy as manifests in their signing of the Milan Food Policy Pact and the hosting of the international horticultural festival of the Floriade. Furthermore, the municipality of Almere formulated a goal to produce 20% of the food they consume themselves (Almere 2009; van der Gaast et al. 2020). One of the means towards this goal is a new neighborhood: Almere Oosterwold. Located at former agricultural land, residents are expected to produce food on their plots. The idea for this neighborhood emerged through a co-creative process with researchers, citizens and municipal agents. Eventually, this idea culminated into a real-life neighborhood which was realized in 2016. Currently, about 2000 residents are living in Oosterwold (Jansma et al. 2010; Jansma and Visser 2011; Brons et al. 2022).

The planning for Oosterwold was unusual. Residents organize the ongoing development of their neighborhood. They organize their own plots as well as the neighborhood including public spaces and basic infrastructure. In the Netherlands, usually the municipality facilitates public infrastructures such as roads, sewage systems, electricity and public spaces such as schools. In Oosterwold, the municipality mainly enforces the rules of Oosterwold, which were written down in contracts that residents sign when buying a plot of land. One of these rules is the mandatory obligation to produce food on 50% of the plot (Jansma and Wertheim-Heck 2021, 2022). However, in the zoning agreement the municipality formulated the ambition for Oosterwold to produce 10% of the total food consumption of Almere (Almere and Zeewolde 2013). In practice this means half of the ambition of the city of Almere, to produce 20% of total food consumption within the city, must be met through urban agriculture in Oosterwold. Yet, the municipality did not specify how this ambition should or could be achieved, leaving open how it can be accomplished by the residents.

### Urban agriculture, entrepreneurship and everyday lived experiences

In this study, we focus specifically on the organizing process of urban agriculture in Almere Oosterwold. With urban agriculture, we mean the production, processing and distributing of food products and services located within or on the fringe of a city (Jansma and Wertheim-Heck 2021). We understand this organizing process of urban agriculture in Oosterwold as entrepreneurial. We define entrepreneurship in this paper as a process of ongoing, creative (re)organizing that intervenes in everyday life (Steyaert 2007; Verduyn 2015). Because it intervenes in everyday life, entrepreneurship is not restricted to commerce and economic drive alone but refers to an overall process of change and transformation. This means the everyday lived experiences of citizens and processes of entrepreneurship are not separate but intertwined (Steyaert and Katz 2004; Steyaert and Hjorth 2006).

Our understanding of entrepreneurship follows the processual understanding of organizing. A non-processual understanding of entrepreneurship understands entrepreneurship through fixed categories, such as firms and products. A processual understanding considers these fixed categories in practice as always in motion (Hjorth et al. 2015). Therefore, organizations such as firms are temporary instantiations within an “underlying sea of ceaseless change” (Nayak and Chia 2011, p.284). They are continuously under construction, emerging, evolving or terminating over time (Langley et al. 2013; Sandberg et al. 2015; Cloutier and Langley 2020). Furthermore, the act of organizing itself is ongoing, which means it is always currently happening, shaped by changing past experiences and future ambitions of the actors that enact them (Schultz and Hernes 2013).

From a processual point of view, entrepreneurial processes intervene in the everyday lived experiences of communities such as Oosterwold because organizing involves practices of everyday life (Langley et al. 2013). In their day-to-day life, members of a community such as Oosterwold enact practices such as cooking and shopping but also producing and selling food. These practices of everyday life are organized by planning and accomplishing them, which involves integrating different tasks and putting them in a certain order (Orlikowski and Yates 2002; Geiger et al. 2020). This means time and temporal coordination is crucial. A good illustration for food entrepreneurship is provided by Cucchi et al. (2021). Practices of everyday life take time to unfold. For instance: it takes time to learn how to produce food. Practices also have a temporal order. For example, before you can sell food, you need to have produced food. In sum, by enacting and organizing their everyday practices, social actors create different temporal situations that in turn enable and constrain how such practices are enacted and organized in the future (Orlikowski and Yates 2002).

To make this more specific, lets illustrate how this works for Oosterwold. Residents of Oosterwold are involved in an ongoing process of co-creating the neighborhood as part of daily life. For this, they enact practices such as running a household, raising kids, having jobs, family obligations, hobby’s. At the same time, they also need to organize their plot and organize urban agriculture. Research in Oosterwold shows there is a temporal order between different practices involving the organization of urban agriculture. Three stages of urban agricultural organizing can be distinguished in Oosterwold based on our findings and an online survey that was conducted in 2020 in Oosterwold (Jansma et al. 2020). In the first stage, residents are *organizing their plots*. They don’t live there yet or in temporary housing while they await the construction of their house. Based on the survey, about 15% of residents is in this stage. In the second stage, residents are *organizing the production of food*. Residents do not produce food or in very small quantities and are mostly concerned with how to produce well, and less with what to do with the food itself. About 45% of the residents is in this stage. In the third stage, residents are organizing *the consumption of food.* This stage is reached when residents produce a surplus. This requires organizing the food that is not consumed by the household, for example through selling, trading, or giving it away. Approximately 40% of residents is in this stage.

These stages do not imply there are stable trajectories in urban agriculture over time. Yet they provide bearings in the temporal flux of ongoing movement (Hjorth et al. 2015) by showing insight into the different temporal situations that residents are in whilst organizing urban agriculture. The process of urban agriculture involves all kinds of activities of different residents, from building and designing their own house to gardening, producing, processing or selling food, all with their own pace and rhythms. This results in a complication: enacting the present out of the imagined future is difficult when there are differences between social actors in terms of the duration and temporal ordering of practices. Furthermore, residents don’t necessarily have the same desires and expectations of the future. Such future imaginaries are shaped by personal circumstances (Mandich 2019; Welch et al. 2020; van der Gaast et al. 2022b). Different future imaginaries in turn incite a negotiating process to converge social actors (Kaplan and Orlikowski 2013; Geiger et al. 2020). This negotiation process is what this paper will explore, a process where we engage residents of Oosterwold to both imagine futures as well as reason back from those futures to the present.

## Methods

### Action research and futuring

In this study, we use an action research approach. With action research, we mean a “collaborative production of scientifically and socially relevant knowledge, transformative action and new social relations through a participatory process” (Wittmayer and Schäpke 2014, p.484). This form of action research is useful in this instance because it is a situation where it is important to not just understand but also stimulate sustainability transformations (Miller 2012; Miller et al. 2014; Wittmayer and Schäpke 2014; Horlings et al. 2019). The food system specifically requires social and organizational change which action research can provide (Braun et al. 2021). As Coghlan and Shani (2020) argue, action research results in organizational change, actionable knowledge and engaging people in a collaborative process all at the same time.

Futuring is the specific method for action research that is chosen in this paper. Futuring refers to the engagement of actors with the future by creating and identifying images of the future in a possibility space for action (Hajer and Pelzer 2018; Oomen et al. 2021). Futuring is argued valuable in food system transformations because it provides a transformative space for both imagining what actions must be taken and what uncertainties can be encountered (Hebinck et al. 2018; Mangnus et al. 2019). Futuring and action research go well together, as the latter “builds on what has taken place in the past, intervenes in the present with a view to shaping the future” (Coghlan and Shani 2020, p.2).

We follow the distinction of Miller (2012) between a knowledge-first approach where action researchers provide knowledge to inspire action, and a process-oriented approach where action researchers actively intervene by facilitating and participating in a process of change. In this study, we choose the latter approach. We use futuring as a deliberative platform where a specific group of citizens, i.e. residents of Almere Oosterwold, can collectively envision imagined futures and reason back from these futures to the present-day. This allows us to explicate two hitherto implicit notions of urban agriculture in Oosterwold. First, what perceptions of the future of urban agriculture exist in Oosterwold? Second, how are those futures realized in the present? On the one hand, we play a facilitative role where we create a deliberative platform to help citizens. On the other hand, we also learn from the process itself through which citizens deliberate. This helps to provide a helping hand to spur a deliberation process regarding the future of urban agriculture where none was in place so far. At the same time, learning from a specific entrepreneurial process within the unique situation of Oosterwold also provides useful scientific insights.

### Visioning, scenario building and backcasting

We explored preferable and possible futures (see Table 1). We used visioning (Year I) to explore *preferable* futures. Visioning helps to uncover what futures are desired by participants and within certain communities (Miller et al. 2014; Mangnus et al. 2019). This method inspires desirable actions and provide directions in terms of the future. We applied scenario building (Year II) to explore *possible* futures. Scenario building enables anticipating the future by plotting desirable directions within a framework of uncertainties (van ‘t Klooster and van Asselt 2006; Miller et al. 2014). Our scenario method was facilitated by a scenario-axis. This axis plots four possible scenarios for the future by selecting two driving forces: current developments visible in society that are most impactful and uncertain. By imagining two extreme states of these driving force, four quadrants emerge that show four possible futures. In this study, visioning and scenario building were combined with backcasting, a technique to trace the future back to actions in the present (Voros 2006; Mangnus et al. 2019). We aimed for the identification of specific actions and actors at the end of backcasting, that allowed the participants to directly act after the sessions were completed.

### Research process and the role of the researcher

**Table 1:** Overview of futuring methods, sessions, participants and data. * = registered participants

Year	Futures	Futuring method	Sessions	Date	Participants	Location	Data sources
I	*Preferable futures*	Visioning and back-casting	1	6-10-2020	56*	Zoom (online)	Recording Zoom and online tools, field notes
			2	17-11-2020	91*	Zoom (online)	Recording Zoom and online tools, field notes
II	*Possible futures*	Scenario method and back-casting	3	21-9-2021	12	Oosterwold (in person)	Drawings of scenario’s, field notes
			4	5-10-2021	12	Oosterwold (in person)	Post-its, field notes

Wittmayer and Schäpke (2014) distinguish several roles for researchers in action research: the reflective scientist is detached and analyzes and reports what happens. A self-reflexive scientist is reflexive of its own role. A knowledge broker tries to make knowledge in terms of sustainability accessible to stakeholders. A process facilitator hosts the deliberative platform but does not participate itself. Lastly, a change agent also seeks to motivate participants to seek change outside the facilitated process. We agree with Horlings et al. (2019) that in practice the boundaries between these roles are blurry. During the research itself, we conformed mostly to the role of facilitator. Yet, even though we did not participate ourself, we did encourage participants to identify specific actions they could take in the present to arrive at their desired futures. In that sense, the role of facilitator slightly overlaps with that of change agent in this case. To illustrate our role, we will discuss how we set up the research process.

First, to initiate our action research process we had to involve (future) residents of Oosterwold. A starting point was a survey which two of the researchers of this paper had conducted in a previous study. Through this survey, the researchers obtained insights in the current state of urban agriculture in Oosterwold. We presented the findings of this survey to the residents of Oosterwold that had participated in this survey. This presentation was used as an opportunity for a first preparatory futuring session. Second, with the momentum of this first meeting, we organized three more meetings (see Table 1). In all meetings, the role of the researchers as facilitator was thoroughly explained. Our role was to facilitate the deliberation process by offering the tools and methods to do so. We set the agenda and the schedules for the workshop, following our futuring method. Yet, in terms of content we did not steer the conversation, nor did we identify or divide tasks or actions as emerging from the backcasting exercise. For the participants, the sessions were useful because they did not had the opportunity yet to collectively discuss possible and preferable futures for urban agriculture. In all sessions, an workshop-atmosphere was created. In the online sessions, we would work in break-out sessions with Murals. In the offline sessions, we would work with professional artists and empty canvases and sticky notes. To make sure all residents had an equal shot at participating, and not just the ones that participated in the first survey, we made online registering forms in advance to each session and spread these forms through email-newsletters of local neighborhood groups as well as through local Facebook groups.

In the next two paragraphs, we will discuss more in depth how these sessions were conducted and how the findings were analyzed.

#### Sessions 1 and 2: preferable futures through visioning

In 2020, we conducted two sessions to explore preferable futures. Due to covid-19 constraints, which impeded face-to-face meetings, these sessions were conducted online. Session 1 was a preparatory meeting where findings were presented of a survey on urban agriculture in Oosterwold. Participants reflected on these findings based on their own experiences. This provided some first insights, and allowed building a relationship with the participants. Session 2 consisted of visioning and backcasting. The visioning started with seven pitches by citizen-participants, in which specific ideas for the future of urban agriculture in Oosterwold were presented. Next, these pitches were the topic of a collaborative backcasting exercise. We used Mural to facilitate this: an online tool that provides a digital canvas with sticky notes. On the top right-hand side of the canvas, the future was symbolized in the form of a specific idea from one of the pitches. On the top left-hand side, the present was symbolized in the form of an empty sheet. Participants were invited to think of specific actions to materialize the imagined future and what actors were expected to perform these actions. Both were written down on virtual sticky notes. The goal was to make a chain of actions from the future to the present, resulting in an action-agenda.

#### Sessions 3 and 4: possible futures through scenario building

In 2021, we used scenario building to plot possible futures. The (temporary) suspension of the lock down allowed for two face-to-face sessions. We selected driving forces in advance through six interviews with residents. In Session 3, we used floor-tape to make an image of this axis on the ground. Participants could literally walk through possible future worlds. Due to time constraints, we only explored two scenarios in detail. We asked participants which scenarios they wanted to explore. In exploring the two scenario’s, participants imagined what would change for urban agriculture in the scenarios. This process was facilitated by an empty canvas, sticky notes and a professional artist that made drawings of what was discussed in real time. Each session was moderated by one of the researchers. The artist afterwards merged the drawings into one image for each scenario. In Session 4, we used these images to back-cast towards the present. We asked the participants what elements they thought desirable in these scenarios, and what actions were demanded in the present to realize this. This was facilitated by a timeline from the present towards the future (2030) that showed fictional news article headlines for events associated with these scenarios. We asked participants to reflect on these events, to identify desirable and undesirable elements and to add and/or rearrange the timeline. Finally, we created an action-agenda with actions and actors that would be useful in both scenarios.

### Data analysis and abductive reasoning

**Fig. 1:**
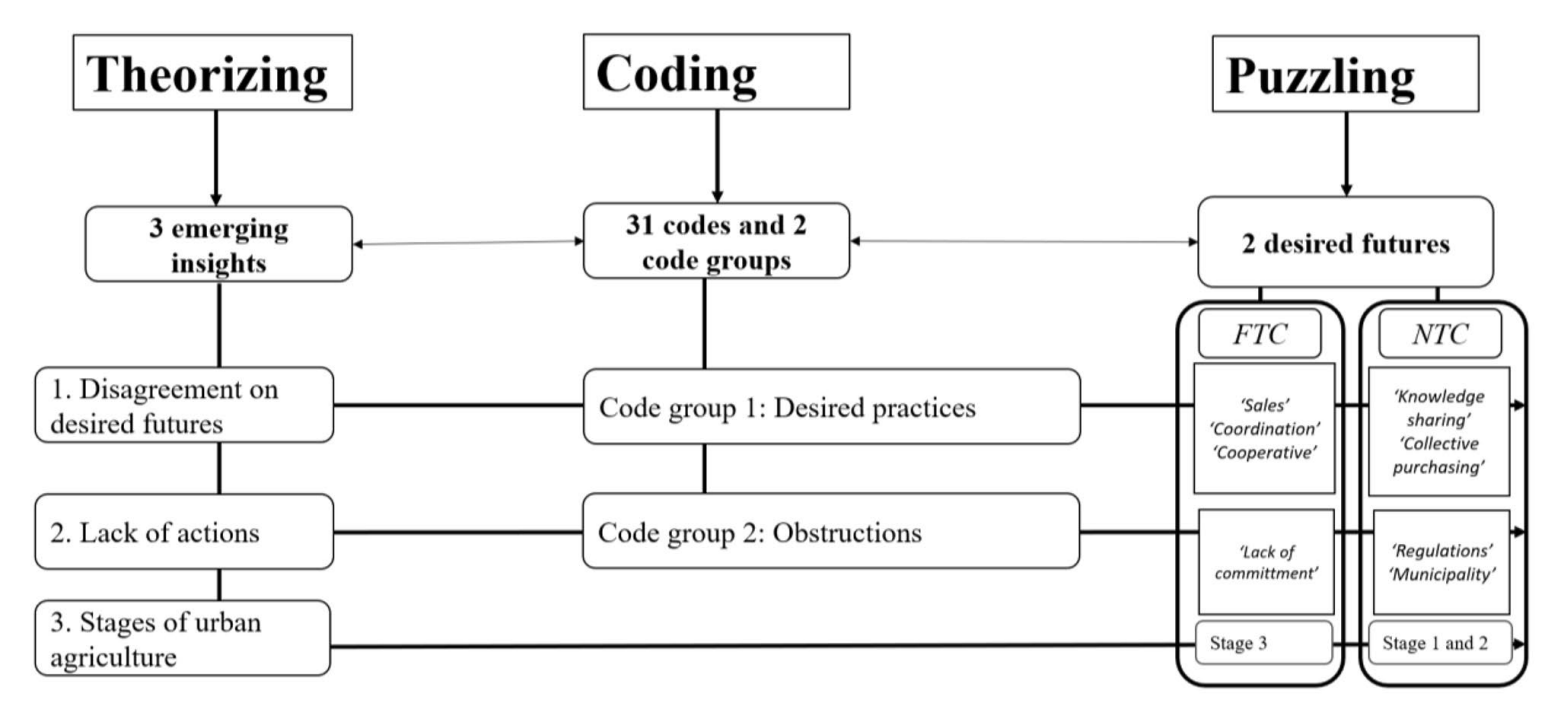
Schematic overview of data analysis and abductive reasoning. Theorizing, coding and puzzling and its results are part of an iterative process (depicted by the two-sided arrows that connect the emerging insights, codes and code groups and the resulting desired futures). The emerging insights (on the left), mediated by the code groups (in the middle) resulted in the identification of two desired futures: FTC and NTC (on the right)

The data analysis of this paper is informed by abductive reasoning. This approach to analysis is often used in action research which involves surprising, unexpected and puzzling experiences. Abductive reasoning helps to understand what is going on whilst being part of the process. Furthermore, it helps to relate these experiences within action research to a larger research context (Shani et al. 2019; Coghlan and Shani 2020). We follow the approach to abductive data analysis as prescribed by Timmermans and Tavory (2022), which we will summarize as an iterative process of theorizing, coding, and puzzling (see Fig. 1).

*Theorizing*, took place during the research process and the futuring sessions. The processual understanding of entrepreneurship was used as an heuristic tool to focus our observations. This resulted in three emerging insights. First, the disagreement between residents with regards to their desired futures. Second, the notion that few participants were willing to take up a role in accomplishing the tasks they identified as needed to arrive at their desired futures. Third, we observed a possible relation between desired futures, unwillingness to act on these futures and the different stages of urban agriculture.

*Coding* was done after the futuring sessions. This was done without the input of participants. For this, the output of the four sessions, which resulted in a variety of data sources (see Table 1), were transcribed to plain text, and inserted into Atlas TI. Following Timmermans and Tavory (2022), we first used open coding for a close reading of our observations. This was done by coding ‘in vivo’ and enlisting interesting observations and statements of participants. This was followed by focused coding, in which similar statements and observations were bundled into new codes. This resulted in 31 codes in total. Looking at the codes that were most grounded (e.g. most recurring in the data), two code groups emerged. Code group one represents codes of desired urban agriculture practices or organizations as emerging from the futuring sessions. Such as: “physical space”, “coordination of produce”, “sales”, “knowledge sharing”, “food production”, “cooperative”, “collective purchasing”. Code group two incorporates codes that represent the factors identified in the obstructions to the taking of desired actions, such as “regulations”, “municipality”, “lack of commitment”.

After coding, the *puzzling* started. By connecting codes and emerging insights, we explored what our study is a case of to come up with an explanation for the puzzling experience of having different desired futures but not much willingness to commit to these futures. By puzzling with the codes, we found similarities between desired actions, obstructions, and different phases of entrepreneurship residents were in (as explained in the conceptual and contextual section of this paper). On the one hand, the residents in stage 1 and 2 of urban agricultural development in Oosterwold were more prone to envision means for “knowledge sharing” and “collective purchasing” because they were not yet able to produce food, and identified “regulations” and “municipality” as a factor that impeded development. On the other hand, the residents in stage 3 emphasized “sales” and “coordination of produce” and were aiming for a “cooperative” to ensure this. Furthermore, these residents considered “lack of commitment” the main issue. This puzzling in turn resulted in two contrasting desired futures: Feeding the City (FTC) and Nourishing the Community (NTC) which we will present in the next section.

## Findings

### Preferable futures: feeding the city vs. nourishing the community

We distinguish two competing perspectives on how the future of urban agriculture should preferably unfold according to the residents of Oosterwold. The Feeding The City (FTC) perspective understands the goal of urban agriculture in Oosterwold as sourcing the city of Almere. This perspective follows the ambition of the municipality to source 10% of total food consumption in the city through food production in Oosterwold. Proponents of the FTC perspective consider this not an exact goal but a guideline to strive towards. Therefore, they see a need for upscaling to produce a surplus that can be distributed to the city. Since the scale of production so far in Almere Oosterwold is insufficient, coordination is required to organize both the production and consumption of food in Almere Oosterwold. In the FTC perspective, food production in Oosterwold can only be called urban agriculture when the broader population in the city of Almere can profit from the produced food. The Dutch word “verwaarden” was often used, which can be translated as adding value. In practice, this means the food that is produced in Oosterwold must find its way to the citizens in Almere. This can be done through idealistic means (e.g. free food for people with less income), or through sales of produced or processed food to local vendors and retailers.

The Nourishing The Community (NTC) perspective in contrast considers the goal of urban agriculture in Oosterwold to facilitate the development of a community. The purpose of urban agriculture is to connect citizens by engaging in a shared activity, not to turn citizens into professional farmers. Therefore, sourcing the city is not a goal but a possible positive side-effect of the development of the community. The NTC perspective considers the 10% target unrealistic and questions the need to upscale which can discourage residents from developing urban agriculture on their plots. By making small steps over time instead, the positivity about urban agriculture can be maintained whilst strengthening the community through shared gardening experiences and activities. Furthermore, upscaling is considered economically unsound. Inexperienced urban agriculturalists must compete with professional farmers on the global market where the prices for bulk products are low. Instead, Oosterwold must target niche-products that don’t require large scale production. Some participants in the NTC perspective reject the notion of commercialization of their produce altogether. Others are pragmatic, they consider selling and processing produce an attractive option to cut costs or even to be able to work less in their day job over time.

Though fundamentally distinct in their vision, the supporters of the FTC and the NTC perspective express similar needs to accomplish their preferable futures. First, they wish for a shared knowledge infrastructure. Upon arrival in Oosterwold, most new residents are unprofessional hobby agriculturalists. To perform agricultural activities, knowledge and skills are required ranging from soil maintenance to crop rotation and equipment use. Simultaneously, the organization of consumption (e.g. trading, selling and/or processing food) requires specific skills and knowledge of regulations with regards to food quality and safety. Therefore, participants agree on the need for an accessible knowledge infrastructure in Oosterwold. Second, participants want shared spaces in Oosterwold for activities ranging from horticulture to processing, and sales. Oosterwold consists of individual plots of residents where residents have to allot space for all their needs and activities such as housing and gardening. There is little space left for urban agricultural activities such as storage or processing. By creating shared spaces, residents don’t have to perform all activities on their own plots. Third, participants agree on the need for shared organizing of the coordination of production and consumption. They see a similar need to coordinate what is produced, in what quantities and by whom.

Yet, the supporters of the FTC and NTC voice different forms of organizing to make sure these needs would be met. Supporters of the FTC perspective propose one organization to meet all needs: a producers cooperative called Cooperative Oosterwold. This organization existed before the futuring sessions. Participants imagine the cooperative will provide seeds and equipment as well as knowledge and skills on how to produce food. Furthermore, the cooperative will organize the logistics by opening a shared space in Oosterwold. This space will function as the central hub for processing, sales, and transport to retailers in the city. As a first step, it is suggested to develop an app to coordinate who produces what crops in what quantity, and to match this with consumer demand in the city.

In contrast, the NTC supporters imagine a wider range of organizations. For the coordination of production and consumption, a consumer cooperative is proposed called VoKo. Since most residents do not yet produce enough to be self-sufficient, they need to procure food from elsewhere to complement their own production. VoKo coordinates this food procurement. It can help residents in Oosterwold that don’t produce enough yet to buy from residents that have a surplus. But VoKo also facilitates collective purchases of organic food from local farmers for a reduced price. Besides VoKo, NTC supporters imagine shared spaces, such as shared fruit processing plants, shared ovens for baked goods and a shared market to sell goods. Other ideas are to build collective greenhouses to produce crops all year round. For the knowledge infrastructure, there is a range of specific fields of expertise in terms of the production of food, from vertical farming to permaculture.

In summary, the FTC and NTC perspectives display a different outlook on what role urban agriculture plays in Oosterwold, and how it should develop. This results in similar needs (i.e. coordination of production and consumption, shared spaces and knowledge infrastructure) with different forms of organizing to meet those needs.

### Possible futures: Manhattan with rules vs. room for everyone

Before presenting the possible futures as imagined by the residents, we explain the driving forces that were chosen. *Regulation* means the municipality will actively monitor for compliance of the rule to use 50% of the plot to produce food. *Self-organization* in contrast means residents are themselves responsible for developing urban agriculture. *Open landscape* refers to a current rule in Oosterwold that residents cannot close off their plots for other residents. Residents must be able to cross the plots of others. *Closed landscape* in turn refers to a current trend: a new area in Oosterwold is now in development which will contain high rises to accommodate more civilians per square meter which will possibly close down the landscape.

#### Manhattan with rules

**Fig. 2:**
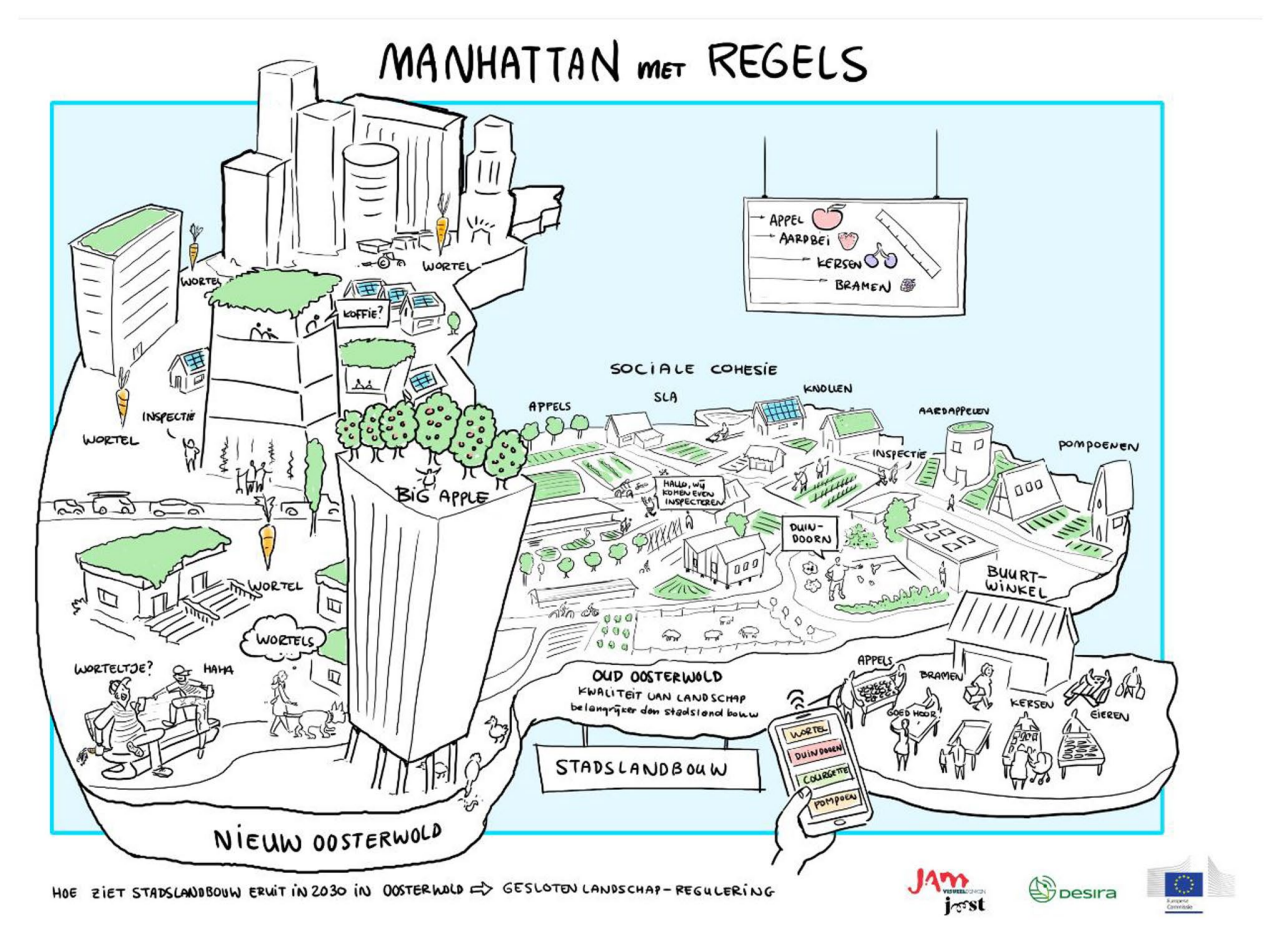
‘Manhattan With Rules’-scenario. With ‘new Oosterwold’ on the left and ‘old Oosterwold’, on the right. Source: Jam Visual Thinking

In the scenario ‘Manhattan With Rules’, urban agriculture will be *regulated* and Oosterwold will have a *closed landscape*. Participants distinguish in this scenario ‘old’ Oosterwold from ‘new’ Oosterwold. ‘Old’ Oosterwold refers to the current situation of several smaller plots with a large diversity in types of houses, and types of produce. ‘New’ Oosterwold on the other hand consists of uniform flats and agricultural monoculture. This is because new residents will, under pressure of increased regulations, hire professional farmers to produce for them. The name ‘Manhattan’ is chosen by the participants because of its association with high-rises. Furthermore, the size of the plot that is under development is roughly the same size as Manhattan. Figure 2 shows this in detail. On the left, in ‘new’ Oosterwold, carrots (‘wortels’ in Dutch) are produced in between the flats in large quantity. On the right, ‘old’ Oosterwold is visible with its higher diversity in types of houses and produce (e.g. ‘aardappelen’ and ‘duindoorn’, potatoes and sea buckthorn in Dutch). In between, there are municipal agents inspecting the compliance of the rules (‘inspectie’, in Dutch).

#### Room for everyone

**Fig. 3:**
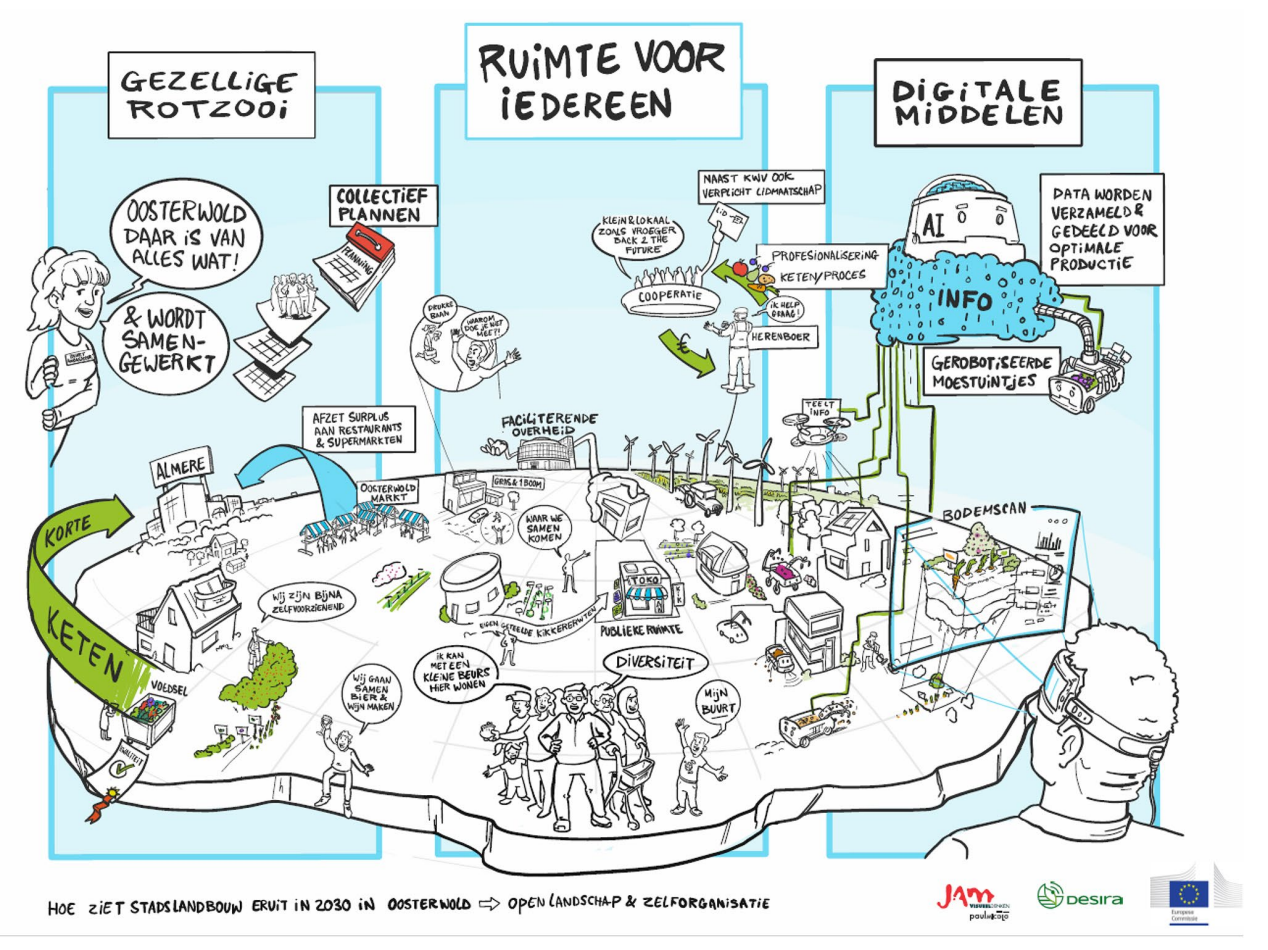
‘Room for Everyone’-scenario. There is both room for sourcing the city (on the left), as well as for people that are too busy to participate (in the middle). Source: Jam Visual Thinking

The Room For Everyone scenario shows an *open landscape* with *self-organization*. In this scenario, there is no divide between ‘old’ and ‘new’ Oosterwold. The name of the scenario signifies the existence of a diversity of types of produce and production methods. As Fig. 3 shows, in this scenario residents produce directly for Almere through short supply chains (‘korte keten’ in Dutch): the produce of residents will source the city of Almere. There is also literally room in the picture for those not willing or able to participate. In the middle, an angry man is yelling ‘why are you not participating?’, to a woman looking at her watch and replying ‘busy job’. This dialogue is embedded in a picture of the stereotypical non-compliant resident with only grass and one tree (‘gras & 1 boom’ in Dutch). Despite the emphasis on self-regulation, diversity and freedom, the scenario also mentions a mandatory membership (‘verplicht lidmaatschap’) of the cooperation that sources the city of Almere.

### Backcasting possible and preferable futures

#### Backcasting preferable futures

During the backcasting session of *preferable* futures, participants explored how the coordination of production and consumption, shared physical spaces and a shared knowledge infrastructure could be realized. Collectively, participants traced the future back to actions in the present. However, individual participants were unable or unwilling to commit to these actions themselves.

The coordination of production was explored through the aspired producer cooperative called Cooperative Oosterwold. During the session, a detailed plan emerged. First, an app must be developed to coordinate the produce and to make sure residents produce according to the demand of the city of Almere. Second, a plot must be procured to collect, process and package the food before it would go to the city. The backcasting exercise halted when the willingness of participants to commit as a member of this cooperative was discussed. The cooperative requires a sufficient amount of residents to become a member to function in practice. A membership means residents need to commit to distribute (part of) their produce through the cooperative. Furthermore, the decision what to produce and in what quantities will have to be based on the demand of the cooperative. The participants were in doubt whether they would be willing to commit to this. They saw value in coordinating who produces what, to avoid ending up with surpluses of one particular product. Especially those participants that were still planning their garden were very interested in aid of the cooperative in deciding what to plant. However, some participants considered the ambition too high. Few residents can already produce in high enough quantities to be interesting to meet the demand of procurers in the city. Also, participants feared losing their independence when they have to adjust what they grow to the demand of others.

The coordination of consumption was explored through the aspired consumer cooperative called VoKo. Prior to the session, the frontrunner of the VoKo had set up a digital infrastructure to coordinate shared consumption. This infrastructure was copied from of a similar initiative he was a member of when he still lived somewhere else. Yet, he didn’t take any further action before having included other residents of Oosterwold since he wanted a ‘critical mass’ of residents to take the next step. The backcasting session provided an opportunity for this. During the session, actions to realize the VoKo were identified. Residents must be recruited that pay a small fee and help with packaging and distributing the food. Also, a pick-up point and administrative tasks must be set up. Moreover, funding, as well as support and awareness in the neighborhood must be raised. However, none of the participants in the backcasting sessions were willing to commit themselves to these tasks. Participants refused to commit because they lacked the time and affinity with organizing and were primarily interested in gardening.

In the case of shared physical spaces, participants in Oosterwold identified actions that require changing regulations. First, procuring a plot without a residential purpose. Now, the only way to buy a plot of land is by building a house on it which complicates organizing a shared space for urban agricultural purposes. Second, building greenhouses on the mandatory 50% of space allotted to urban agriculture. Now, greenhouses are regarded construction and not agricultural food production. This means they are only allowed on the part of the plot designated for home construction. Constructing greenhouses on a plot limits the space for living. Therefore, according to the participants, the first action for achieving shared spaces must be taken by the municipality.

For a desired knowledge infrastructure, a different reason emerged for the municipality to take the first step. The participants mentioned there is a lot going on in Oosterwold already regarding knowledge sharing. Participants discussed the plethora of knowledge sharing initiatives in Oosterwold they already knew that all more or less had the same objectives. Some argued that especially with regards to the information technology infrastructure (e.g. hosting and managing websites and apps), concerted action would avoid individuals inefficiently re-inventing the wheel on individual pursuits, while harnessing collective knowledge and experience. A collective organization overseeing and merging the separate individual initiatives was argued more efficient and encouraging. The participants argued the municipality is responsible for facilitating such collective infrastructures. The costs and efforts of setting up such a central knowledge infrastructure should not fall on one or more individuals when the benefits go the community as a whole.

In sum, residents identified necessary actions but were unable or unwilling to take those actions themselves. In the case of shared physical spaces and a shared knowledge infrastructure, the municipality was singled out as the actor to take these actions.

#### Backcasting possible futures

In backcasting *possible* futures, participants first identified the desirable elements in the scenarios, and next articulated the requirements to realize these desirable elements. The participants identified a set of actions but similar to the session on backcasting preferable futures they did not commit themselves to the actions. Instead, they pointed to both the Cooperative Oosterwold and the municipality as the actors that should take the first step.

In discussing desired elements in the possible futures, the contrasting visions of Feeding the City (FTC) and Nourishing the Community (NTC) were visible. From the FTC point of view, desirable elements in the ‘Manhatten With Rules’-scenario are the inspections of the municipality and the professional farmers that are hired to fulfill the urban agriculture obligations. The inspections enforce compliance with the rules. Either residents themselves make an effort to produce food. Or residents, who cannot or will not do the work themselves, hire professional farmers to produce food on their plots. This leads to producing more food one way or the other, resulting in better opportunities to source the city. Furthermore, the emphasis on sourcing the city of Almere, as well as the crucial role of Cooperative Oosterwold to realize this, is deemed positive in the ‘Room for Everyone’-scenario. In contrast, for the NTC perspective, ‘new’ Oosterwold in the ‘Manhattan With Rules’-scenario was considered the death of Oosterwold as it was intended. For them, the open landscape is more important than upscaling urban agriculture because it helps to develop Oosterwold as a community where everyone connects with one another. They consider the diversity of ‘old’ Oosterwold as the main goal. Similarly, they like the fact that those that have busy lives and only have grass and one tree, still have a place in Oosterwold, as the ‘Room For Everyone’-scenario suggests.

Eventually, a compromise was reached between FTC and NTC perspectives resulting in the following aspired actions. First, professional farmers don’t replace but facilitate residents in food production. For instance, by helping residents to develop skills or assisting in harvesting the produce. It was also suggested more experienced residents should take up mentoring roles for less experienced residents. Second, the inspection of the compliance with food production should not be enforced from the top down, but from the bottom up. Residents inspect each other, though not to sanction non-compliance, but to help co-residents to comply. Third, an obligatory introduction workshop on urban agriculture for new residents should be implemented. Fourth, it was proposed to widen the scope from the development of food production alone to the monitoring of social development as well. It was not completely specified how social development could be measured. Some participants suggested a credits-system where activities that help develop the neighborhood of Oosterwold will be recorded and rewarded. Lastly, the cooperative should maintain flexibility in terms of accepting harvests’ fluctuations, with food production still in development and residents having different levels of experience.

In discussing who should perform these actions, the participants refused to commit individually. They argued the municipality should take the lead, repeating previously voiced arguments in the sessions on preferable futures. First, the participants reasoned the benefits are collective, and therefore the burden should not be on individuals. Second, a municipal agent knows better what is allowed and what isn’t and therefore is better equipped to navigate the complex regulatory dimensions of urban agriculture. The participants exemplified this with the issue of food safety regulations. Residents expressed they were not confident in knowing what is allowed with regards to selling and processing food. Therefore, the participants suggested the municipality should provide an intermediary agent or agency for assistance not only in food production, but also for guidance in navigating regulation. Here, it is relevant to also note that one participant suggested that this intermediary agent could also be hired by the residents themselves by pooling together the required funds. This idea was opposed by most participants because they did not want to assert the financial risk for hiring personnel themselves, despite the fact this risk would be a collective risk.

In sum, participants were able to backcast the desirable elements of possible futures towards specific actions. They were however unable or unwilling to commit to those actions themselves. Instead, they argued the municipality should take the next step.

## Discussion

### Imagined futures, present actions and temporal dissonance in Almere Oosterwold

In establishing the neighborhood of Oosterwold, the municipality of Almere formulated an ambition of producing 10% of Almere food consumption in Oosterwold without specifying how this should or could be accomplished. This paved the way for an entrepreneurial process of organizing food production and consumption which our study has explicated. This paper has two main findings. First, the identification of two clashing future perspectives, respectively FTC (Feeding the City) and NTC (Nourishing the Community). Second, the observation that participants were unwilling or unable to commit themselves to the actions they identified to enact the desired futures in the present. Partially, the different future perspectives can be attributed to different ideas on forehand on what Oosterwold can or should be. Some came to Almere Oosterwold to change the food system, others for gardening and green spaces (Jansma et al. 2020). The former might be more inclined to favor fast upscaling of production, whereas the latter might a favor incremental development of urban agriculture that facilitates community development.

On the other hand, the entrepreneurial process that was revealed through this study, and the temporal ordering within the organizing process, also provides an explanation for these findings. The futuring sessions in this study resemble what Kaplan and Orlikowski (2013) and Geiger et al. (2020) call temporal work, i.e. linking of projections of the future with views of past and present to resolve tensions between different understandings. Yet, these authors also state the negotiation between converging actors and their different temporal structures can be time intensive (Geiger et al. 2020) and does not always lead to organizational change, sometimes it can be obstructed by inertia (Kaplan and Orlikowski 2013). One cause of inertia can be the existence of multiple temporalities between social actors (Bastian and Bayliss Hawitt 2022) which leads to “contradictory expectations about how to temporally structure their activities” (Orlikowski and Yates 2002, p.687). According to Geiger et al. in that situations temporal autonomy is required, which they describe as looking ahead in time. For instance by synchronizing activities and routines. Yet, the findings of this study shows the opposite of this. It resembles what Zivkovic (2018) calls temporal dissonance, it is the “disjuncture experienced by participants in the act of emplacing themselves or their loved ones ahead of time” (Zivkovic 2018, p.20). In this study, temporal dissonance means it is not only hard for participants to look ahead, but also to look back. In other words, it is the inability to look beyond the own temporal situation (e.g. stage of urban agricultural development) one occupies.

To illustrate this, it can be useful to reiterate the three different stages of urban agricultural development in Oosterwold we identified in the conceptual and contextual context in this paper: the organization of the plot (1), the organization of production (2) and the organization of consumption (3). As we have seen in this study, participants were not all in the same stage during the futuring process. Whereas some residents still designed and constructed their houses (1), others were starting food production (2) or already produced a surplus (3). For those residents in (1) and (2), the idea of what to do with the surplus was less urgent. In turn, those that produced a surplus (3) had to deal with the food products they could not consume themselves, and therefore were more concerned with the coordination of production and consumption. Furthermore, whereas some residents progress swiftly through these stages, for others it can take more time and effort. Some encounter more constraints (e.g. time, knowledge, skills) than others to develop urban agriculture and can become ‘stuck’ in one stage. For instance, residents with jobs, kids and other pressing tasks and with less preexisting skills in food production take longer to get to stage (2) or (3), than residents that already are familiar with gardening, are retired and/or have no jobs or families. 

In sum, residents have different lived experiences in Oosterwold because they operate in different stages of urban agricultural development. This leads to temporal dissonance. During the futuring process, residents producing at scale found it hard to understand residents with trouble getting there. In turn, residents that were not producing yet, or only on a small scale, did not feel the pressure of a surplus of food that needs to be organized. Therefore, these different lived experiences resulted in different imagined futures. The stereotypical resident with ‘grass and one tree’ provides an illustrative example. The FTC perspective considers this type of resident an impediment to urban agriculture, imploring inspections for compliance. The NTC perspective in turn stress the ‘split’, between daily life and the goal of urban agriculture and has understanding for this type of citizen, who’s actions can be explained by the lack of time to develop urban agriculture next to a (fulltime) job or a family. Here we can discern the different temporal situations manifesting. Residents that feel the urgency of fast upscaling due to the fact they produce a surplus, versus residents that are constrained by the confines of a busy daily life in developing urban agriculture.

Temporal dissonance also plays a role in the lack of commitment to the identified actions. Despite this lack of commitment, many participants provided examples of themselves organizing what they need as part of daily life. Participants discussed examples such as a baker seeking poppy seeds for the bread he was baking, and a resident supplying him some for free in exchange for a loaf of bread. There were also examples of participants organizing individual processing facilities (e.g. professional ovens). In other words, the participants were not idle. They were indeed putting in the work to develop urban agriculture in their neighborhood. Yet, they did so within the confines of their own temporal situation. Residents that already have produce to spare seek out other residents that have a use for them. Residents that are still struggling with how to produce food find likeminded residents to share experiences and swap skills. This shows residents have trouble in committing to actions they themselves don’t directly profit from (yet). This again highlights the role of temporal dissonance, i.e. the myopia of participants to look beyond their own temporal situation. Participants did not look back or ahead in organizing what is needed to develop urban agriculture, they just committed to organize what they required themselves in the present.

### Methodological repercussions of temporal dissonance

In the previous paragraph, we presented temporal dissonance as an explanation of why contrasting desired futures emerged and why participants were unable or unwilling to commit to the actions they identified. This is in line with our action-research approach which arrives at an exploratory hypothesis of what was going on in the action research process (Shani et al. 2019). This begs the question what can be learned from this explanation for similar studies. Braun et al. (2021) presents an action research study of 22 enterprises in farming, processing and trading in the region of Berlin, Germany. In this study, they facilitate an interorganizational learning process for entrepreneurs to both learn from one another as well as to strengthen the local supply chain. This study manages to incite actors to question routines and foster new visions. In contrast, in Oosterwold most participants were hardly experienced in food production and almost none were professional entrepreneurs. Furthermore, in our study they were asked to come up with shared visions for the neighborhood, instead of individual visions for their own enterprise. Residents were invited to design a future of urban agriculture collectively which resulted in clear plans of action for formalized, collective organizations. Temporal dissonance complicated this attempt.

Mangnus et al. (2019) involves a futuring study that includes both visioning and backcasting in the context of sustainable urban food systems in Japan. In this study, a clear set of imagined futures and present actions are presented. Yet, the study also mentions time constraints in this study. These constraints make it impossible to assess the impact of these futures and actions. Since this study was not conducted through an action-research approach, the insight into the process through which imagined futures and present actions were organized was absent. Our study showed futuring can be hindered by temporal dissonance when combined with an action-research approach. Therefore, it demonstrates futuring does not generate change out of nowhere. When the imagined futures don’t match the lived experiences of citizens they will not materialize. However, it is important to mention that the sessions are a snapshot of the ongoing development of urban agriculture in Oosterwold. It continued after our last session and still continues. Therefore, it is hard to claim with certainty what our study did, and did not incite in terms of change.

In sum, our study shows the temporal context matters in futuring and action research in organizing processes such as entrepreneurship. In our study, residents of Oosterwold shared a similar spatial context but at the same time they were occupying different temporal situations. Therefore, future research should take the temporal context into consideration. In this study, this was done by exploring in advance the population under study through a survey (Jansma et al. 2020). This helped to already have an insight into the different stages of urban agriculture, and how this affects the visions of residents. However, even though this survey provided useful background information, it did not provide a thorough sociological assessment of how the community of Oosterwold functions. Therefore, future studies could also incorporate a study of the pre-existing social ties in the community. This helps to understand the relationship between strong and weak ties with temporal dissonance and its effects on entrepreneurship.

### Entrepreneurship and urban food planning

In the introduction, it was mentioned studies of urban food planning seldom explore the role of entrepreneurship vis-a-vis planning (Morgan 2010; Opitz et al. 2015; Sonnino and Coulson 2020). This study has showed more insight into this. As explained before, the municipality of Almere specifically designed the neighborhood of Oosterwold, including urban agriculture, with limited government planning. They deliberately did not plan for a public enterprise or service for urban agriculture. Currently, there are no official processes and places where residents regularly (can) meet and discuss their progress and problems with urban agriculture. However, the municipality did formulate an ambition for the food production in Oosterwold to be enough to source the rest of the city of Almere as well. Therefore, they expected limited planning would result in the residents of Oosterwold organizing both the production and consumption of food themselves. In other words, the municipality expected that limited government planning would result in entrepreneurship.

This study shows limited government planning can result in entrepreneurship. Residents of Oosterwold make efforts to organize production and consumption of food. However, residents also encounter obstacles and limitations for which they claim the government is best suited to remove them, such as the regulations regarding a shared physical space and food safety issues. Some residents already try to navigate regulations inventively. For instance, residents worked around the rule that makes it impossible buy a separate plot just for the purpose of a shared physical space. They asked permission to use an empty plot that cannot be used for construction because it contains an archeological significant site. Residents are also in contact with the municipality to change the rules regarding the building of greenhouses, in order to make it count as part of the mandatory urban agriculture efforts. Yet, all these efforts require skills, time, and resources, and continuous efforts. Not all residents have these to their disposal in equal share. This explains the wish of residents that the municipality hires an intermediary to guide their entrepreneurial process and help them where they themselves lack the time or the skills.

 In sum, even though an entrepreneurial process has emerged where government planning was deliberately limited, further development requires more government planning than has hitherto been provided. If the municipality does not step in to provide more government planning, it is possible residents of Oosterwold are discouraged altogether. This could lead over time to the downplaying of urban agriculture in the neighborhood, resulting in a failure to meet the aims as posed by the municipality. This shows entrepreneurship and planning are complementary social processes that need one another to succeed.

Based on this insight, it is possible to formulate two recommendations for urban food planning and policy. First, when formulating policy ambitions for sustainable food systems, government actors must at forehand explore to what extent they expect citizens and entrepreneurship to organize this themselves. This way, they can already anticipate the need for (more) government planning in those instances citizens and entrepreneurship cannot do it alone. Second, it is important to strengthen the role of intermediary actors by government agencies, as requested by the residents of Oosterwold. This intermediary actor resembles what Giambartolomei et al. (2021) call policy entrepreneurs, actors both inside and outside the government that foster crucial relationships and networks and thereby not only set things in motion but also inspire other actors to make an impact. In short, by looking ahead and hiring intermediary actors that bring planning and entrepreneurship together, the reciprocal relationship between entrepreneurship and planning can be improved.

## Conclusion

Cities increasingly envision more sustainable food futures. The realization of those futures is often understood from a planning perspective, leaving the role of entrepreneurship unspecified. This paper explicates a specific entrepreneurial process to understand how it contributes to realizing sustainable urban food futures. We studied Almere Oosterwold, a neighborhood where residents are contractually obligated to produce food on 50% of their plots. The municipality of Almere has the objective to source 10% of the total Almere food consumption from Oosterwold, yet it did not specify how this goal should be achieved. By using futuring as a methodological platform, we engaged residents of Oosterwold to explore what possible and preferable futures they imagine for urban agriculture in their neighborhood. Following, we backcasted these futures towards concrete actions in the present. Our findings show contrasting future perspectives that underlie the entrepreneurial process. Furthermore, we observe a lack of commitment of residents in realizing imagined food futures. We argue the contrasting futures and lack of commitment are the result of a temporal dissonance; a myopia where residents have trouble looking beyond their own situation. Temporal dissonance relates to the existence of different temporal situations since every resident has its own pace in how urban agriculture develops, which explains the discrepancy between images of the future and what residents can and want to commit to individually and collectively.

Based on this, we conclude imagined futures must fit with the lived experiences of citizens in order to be realized. Citizens have different situations and perspectives on what is desirable which can constrain entrepreneurship. Therefore, planning is required to spur collective action where none emerges on its own. In sum, urban food futures need both planning and entrepreneurship to be realized since they are complementary social processes.
